# RETRACTED: Articles from the Special Issue “Effect of Hot Manufacturing Methods on Material Processing by Finite Element Modelling”

**DOI:** 10.3390/ma19132907

**Published:** 2026-07-07

**Authors:** Materials Editorial Office

**Affiliations:** MDPI AG, Grosspeteranlage 5, 4052 Basel, Switzerland

The journal retracts all published papers in the Special Issue: “Effect of Hot Manufacturing Methods on Material Processing by Finite Element Modelling” 

Following publication, concerns were raised regarding an article in this Special Issue. In accordance with the journal’s established procedures, the Editorial Office and Editorial Board, with support from MDPI’s Research Integrity team, initiated an investigation. This investigation was subsequently extended to all articles published within the Special Issue.

The investigation identified a number of issues affecting the editorial handling and peer-review of submissions. These included undisclosed conflicts of interest between authors and Guest Editors, as well as patterns of citation that did not comply with MDPIs’ citation policies. Based on these findings, the investigation concluded that the integrity of the publication process for this Special Issue has been compromised. As a result, the reliability of the articles included in this issue cannot be assured.

On this basis, the Editor-in-Chief has decided to retract the affected articles [1,2,3,4,5,6,7] in accordance with the journal’s retraction policy and the guidance of the Committee on Publication Ethics (COPE).

Details of the affected articles, including author responses where available, are provided below.

1. **RETRACTED: Memon et al. Analysis of Friction Stir Welding Tool Offset on the Bonding and Properties of Al–Mg–Si Alloy T-Joints. *Materials* 2021, *14*, 3604**

The authors have been informed of this retraction and have indicated their disagreement with this decision.

2. **RETRACTED: Memon et al. Effects of FSW Tool Plunge Depth on Properties of an Al-Mg-Si Alloy T-Joint: Thermomechanical Modeling and Experimental Evaluation. *Materials* 2021, *14*, 4754**

The authors have been informed of this retraction and have indicated their disagreement with this decision.

3. **RETRACTED: Memon et al. Thermo-Mechanical Simulation of Underwater Friction Stir Welding of Low Carbon Steel. *Materials* 2021, *14*, 4953**

The authors have been informed of this retraction and have indicated their disagreement with this decision.

4. **RETRACTED: Ghiasvand et al. Investigation of Mechanical and Microstructural Properties of Welded Specimens of AA6061-T6 Alloy with Friction Stir Welding and Parallel-Friction Stir Welding Methods. *Materials* 2021, *14*, 6003**

The authors have been informed of this retraction and have indicated their disagreement with this decision.

5. **RETRACTED: Chupradit et al. Pin Angle Thermal Effects on Friction Stir Welding of AA5058 Aluminum Alloy: CFD Simulation and Experimental Validation. *Materials* 2021, *14*, 7565**

The authors have been informed of this retraction and have indicated their disagreement with this decision.

6. **RETRACTED: Ghiasvand et al. Investigation of the Effects of Tool Positioning Factors on Peak Temperature in Dissimilar Friction Stir Welding of AA6061-T6 and AA7075-T6 Aluminum Alloys. *Materials* 2022, *15*, 702**

The authors have been informed of this retraction and have indicated their disagreement with this decision.

7. **RETRACTED: Ghiasvand et al. Effect of Tool Positioning Factors on the Strength of Dissimilar Friction Stir Welded Joints of AA7075-T6 and AA6061-T6. *Materials* 2022, *15*, 2463**

The authors have been informed of this retraction and have indicated their disagreement with this decision.
